# Autoimmune Disease Genetics 2013

**DOI:** 10.1155/2014/487643

**Published:** 2014-07-06

**Authors:** Timothy B. Niewold, George N. Goulielmos, Shervin Assassi

**Affiliations:** ^1^Department of Immunology and Division of Rheumatology, Mayo Clinic, Rochester, MN 55905, USA; ^2^Laboratory of Molecular Medicine and Human Genetics, Department of Medicine, University of Crete, Heraklion, Greece; ^3^Department of Rheumatology, University of Texas Health Science Center at Houston, Houston, TX 77030, USA

The familial predilection toward autoimmunity is in part due to genetic risk factors, and recent studies have greatly expanded our understanding of the complex genetic architecture underlying autoimmune disease. Technical advances and the assembly of large patient cohorts have resulted in rapid progress in the field, which does not appear to be slowing. Studies which have included multiple ancestral backgrounds have demonstrated differences in risk factors between ancestral backgrounds, as well as some similarities [1–3]. Also, genetic studies examining subphenotypes in autoimmune diseases have illustrated the idea of biological diversity within a complex condition, that different individuals with a given condition have different genetic risk factors, and some of the clinical differences between patients are likely related to this fact [4–6]. In these ways, understanding the genetic basis of disease provides us with some tools that could eventually be useful in developing more individualized diagnostic and therapeutic strategies, enabling personalized medicine.

In the current issue, a series of papers highlight some exciting topics within autoimmune disease genetics. M. G. Zavala-Cerna et al. explore genetic associations between PAD4 and rheumatoid arthritis in a Mexican cohort. The PAD4 enzyme is implicated in posttranslational protein modification characteristically targeted by rheumatoid arthritis-associated autoantibodies. B. N. Frederiksen et al. examine genetic polymorphisms underlying type I diabetes and islet cell autoimmunity, finding both age- and disease-stage relevant differences in association. This study illustrates the complex ways in which genetic factors can influence disease, and it is likely that this complexity occurs in many different autoimmune diseases. Genetic risk alleles may depend on other factors such as age, disease stage, and environment to influence risk of disease. Discovering these relationships will greatly improve our understanding of autoimmune disease pathogenesis. C. E. Weckerle et al. report a familial aggregation study looking at circulating levels of tumor necrosis factor alpha, a cytokine which is elevated in lupus patients [7], in unaffected members of lupus families. Previous work had demonstrated familial correlation in type I interferon levels [8], and the current study documents a familial relationship in tumor necrosis factor alpha levels. Interestingly, while type I interferon was only correlated within genetically related family members and not correlated between patients and spouses, tumor necrosis factor alpha was correlated between lupus patients and their spouses, suggesting a potential environmental influence on tumor necrosis factor alpha levels.

S. A. Zavaleta-Muñiz et al. study polymorphisms in the IL6 gene with regard to susceptibility to rheumatoid arthritis. A. Zóka et al. provide a comprehensive review of the alterations in the immune system which are related to type I diabetes. H. C. Chai et al. examine polymorphisms in genes within the Toll-like receptor and type I interferon pathways in systemic lupus erythematosus patients from a South Asian population, extending our knowledge of these susceptibility genes to an additional world population. While one issue cannot be comprehensive, the studies included in this issue provide an overview of some of the current frontiers in the genetics of autoimmune disease.

*Timothy  B.  Niewold* *Timothy  B.  Niewold* *George  N.  Goulielmos* *George  N.  Goulielmos* *Shervin  Assassi* *Shervin  Assassi*
